# Family psychoeducation in schizophrenia and schizophrenia related disorder, treatment compliance, and suicidal risk reduction: questions about their relationship from a naturalistic observation

**DOI:** 10.3389/fpsyt.2024.1370566

**Published:** 2024-04-04

**Authors:** Yann Hode, Romain Padovani, Wydad Hikmat, Nathalie Guillard-Bouhet, Jérome Attal, Marie-Cecile Bralet, Mélanie Biotteau, Isabelle Chereau Boudet, Olivier Canceil, Aurélie Montagne Larmurier, Céline Roussel, Stéphanie Lemestré, Dominique Willard

**Affiliations:** ^1^ Association Psychoeducation PROFAMILLE, Chatenois, France; ^2^ Independent Researcher, Le Tampon, Réunion, France; ^3^ Psychiatric Hospital of Kelaa Sraghna, Ministry of Health, Morocco, Kelâa des Sraghna, Morocco; ^4^ CREATIV Centre de REhabilitation et d'Activités Thérapeutiques Intersectoriel de la Vienne, Centre Hospitalier Henri Laborit, Poitiers, France; ^5^ La Colombière, Centre Hospitalier Universitaire de Montpellier, Montpellier, France; ^6^ CRISALID-HDF (Department Support of cognitive remediation and psychosocial rehabilitation- South Hauts de France area), Etablissement Public de Santé Mentale Oise, Clermont de l Oise, France; ^7^ INSERM Unit Research 1247 GRAP, Picardie Jules Vernes University, Amiens, France; ^8^ GDR 3557 Research network, Addiction and Psychiatry, Paris, France; ^9^ Centre Hospitalier Isarien, Clermont de l’Oise, France; ^10^ Centre Hospitalier Universitaire de Tours, Tours, France; ^11^ Centre Expert Schizophrenie, Centre Hospitalier Universitaire de Clermont-Ferrand, Clermont Ferrand, France; ^12^ Fondation Santé des Etudiants de France, Paris, France; ^13^ Sante Mentale France, Paris, France; ^14^ Service de Psychiatrie Adulte, Centre Hospitalier Universitaire de Caen, Caen, France; ^15^ Centre Hospitalier Annecy Genevois (CH Annecy), Metz-Tessy, France; ^16^ Association de psychoéducation des Familles Profamille Liège Belgique, Liège, Belgium; ^17^ Pôle PEPIT (Pôle Hospitalo-Universitaire d’Evaluation Prévention et Innovation Thérapeutique), Groupe Hospitalier Universitaire Paris psychiatrie et neurosciences, Paris, France

**Keywords:** schizophrenia, psychoeducation, suicide, compliance, treatment, family intervention, suicide attempt

## Abstract

**Introduction:**

The Profamille V3.2 multi-family psycho-educational program directed at caregivers of relatives with schizophrenia or schizophrenia related disorder has been shown to decrease the annual prevalence of suicide attempts. It has been reported that psychoeducation of families can sometimes improve compliance with treatment. This study investigates whether the Profamille program improves compliance and thus reduces the risk of suicide among patients.

**Method:**

This is a retrospective study of 179 groups of family caregivers, encompassing 1946 participants enrolled in Module 1 of the Profamille program and followed up one year after completion of the module. Evaluations were conducted using questionnaires filled out by family caregivers at three distinct times: prior to beginning the program, upon its completion, and again one year following its conclusion. The annual prevalence of suicide attempts was measured both before the program began and one year after its conclusion, while compliance to treatment was evaluated at the start and end of the program.

**Result:**

After the Profamille program, the annual prevalence of suicide attempts fell by a factor of 2 (p-value = 0.00002) and patient compliance improved (p-value <0.000001). This reduction in suicide attempts was observed independently of improved compliance. Compliance seems to have an additional effect, but only after participation in the program.

**Conclusion:**

The Profamille program reduces patients' risk of suicide even when patients are not taking the treatment. When family psychoeducation is not proposed in schizophrenia or schizophrenia related disorder, this can represent a loss of chance for patients.

## Introduction

Schizophrenia is a chronic and severe pathology. Its lifetime prevalence varies, ranging from 0.30% to 0.66%, with an incidence of 10.2 to 22.0 per 100,000 person-years (1). The burden associated with this disease is considerable, with severe loss of autonomy and significant comorbidities (2, 3).

Life expectancy is also significantly reduced. Based on the standardized mortality ratio, people with schizophrenia have a risk of death two to three times higher (for all-cause mortality), and this gap in mortality rates has widened over the last few decades (1). In fact, in developed countries, the disease is associated with a loss of nearly 15 years of life compared to the general population, all causes combined (4). Although other risk factors (physical health, co-morbidities, addictions, etc.) should not be ignored, suicide seems to occupy an important place among these lost years of life (5). While data varies, most studies suggest that around 5-6% of people with schizophrenia will die by suicide (6). Regular antipsychotic medication use is often associated with increased life expectancy (2, 3). However, the specific impact of these treatments on reducing suicidal risk is not clearly established, except for certain drugs, in particular clozapine, which have been shown to be effective (7).

The impact of family intervention on reducing suicide risk remains underexplored. A family-based comprehensive intervention (8) paradoxically shows a slight increase in suicidal risk. On the other hand, a cognitive-behavioral approach involving the families of adolescents who had attempted suicide showed a reduction in risk, but only over a three-month follow-up (9). In addition, a retrospective analysis of over 800 family carers taking part in the Profamille psycho-educational program, which uses a cognitive-behavioral method, showed that suicide attempts by patients were halved one year later compared with one year before participation in the program (10).

Several studies point out that psychoeducation of family caregivers could improve patient compliance (11, 12). Data from Hogarty’s team in the early 1990s already suggested this, without providing definitive proof (13). In 2008, a literature review by an Australian research team suggested involving caregivers to improve medication compliance (14). Expert consensus recommends family psychoeducation to improve adherence in patients with severe mental disorders (15).

A negative attitude towards medication is a known risk factor for poor compliance (16). Psychoeducation of the family can convey a more accurate vision and reduce negative stereotypes about treatment (17). However, the effect of family psychoeducation on adherence is not always found (18).

In this article, we examine the extent to which the drop in suicide attempts (SA) observed in the Profamille psycho-educational program is linked to better treatment compliance. We use previously unanalyzed data on patient compliance collected during the study showing a significant drop in suicide attempts in the Profamille program (10). These were complemented with data from groups who, at the time of the first study, had not reached one year’ follow-up after the end of the program’ first Module.

## Method

This is a retrospective study of teams in the French-speaking network of users of the Profamille psychoeducational program. The data presented in this study come from all 47 teams of this network that offered version V3.2 of the program and reassessed participants one year after Module 1. The teams were from Belgium, France, and Switzerland, and offered this program between 2011 and 2019.

### Intervention

The Profamille multifamily psychoeducational program is based on a cognitive-behavioral approach. It is a French-language program that has been available for approximately 35 years but has been improved in successive versions through a continuous process of evaluation. The Profamille program is a routine offering and not a research program. The intervention used the version V3.2. This version consists of 2 modules, an initial training module (Module 1) of 14 sessions at weekly or fortnightly intervals, and a consolidation module (Module 2) of 4 sessions over 2 years. Sessions last 4 hours and follow a precise, highly structured sequence. A group is usually made up of twelve participants and two trained instructors. Anonymous evaluations were routinely carried out in the form of self-questionnaires at the beginning and end of the first module program, as well as one year later. These evaluations are part of the quality assurance criteria designed to measure the effects obtained, guarantee the fidelity of program delivery, and certify the teams. These questionnaires are filled in by caregivers about the condition of their close relative who is ill.

Patients are not present at the sessions, and family members attend independently of their relative’s psychiatric care. Their participation is not conditional on the agreement or information of the patient or his/her physician.

#### Participants

The study was carried out on 179 groups for which follow-up assessments were available one year after Module 1 and involving 1946 participants. Out of these participants, 1193 had data completed one year after the end of Module 1. There was therefore 39% missing data at the end, either due to drop-outs, or to organizational problems encountered by the teams in collecting and entering the questionnaires.

Participants were recruited through an article in a local newspaper or by referral from a family organization or physician. The title of the psychoeducation groups explicitly states “for families with a relative with schizophrenia or a related disorder” and this is clearly explained to families before they are enrolled and to all professionals who may refer families. The participants were all family members of a patient with schizophrenia or a related disorder. Most were the patient’s parents, but some were the patient’s partner or sibling.

Numerous studies have demonstrated that family psychoeducation in severe mental illness, and in schizophrenia in particular, has a significant positive effect on the patient, and is a highly recommended practice. However, to date, only a very small percentage of families benefit from such programs, which means lost opportunities for many patients. In order to reach out to more families and thus help more patients, and to have an adapted offer, inclusion criteria for the program avoid unnecessary constraints. For example, sometimes, the patient’s diagnosis is unknown or not stated because the patient refused to meet a doctor or refused to allow a doctor to provide information to the family. In these cases, the relatives who contact the teams meet a experienced psychiatrist with expertise in schizophrenia to check whether the behavior and history of the patient (treatments, hospitalizations, course of symptoms, …) they are reporting are mainly in favor of a diagnosis of schizophrenia or schizophrenia related disorder and whether the program is suitable for them.

The teams and the psychiatrists associated with them instructed not to recruit family members whose loved ones might have a diagnosis outside the schizophrenia spectrum disorder framework, for a very simple reason: during the sessions, the patient’s symptoms and behaviors are extensively described and analyzed, and a participant whose loved one has symptoms or behavior that are too different will feel uncomfortable, too different from the others and no motivate to continue, risk stopping prematurely and be detrimental to the group dynamic.

The possibility of a diagnosis of brief psychotic disorder, or schizophreniform disorder leads to caregivers’ ineligibility, because the duration of the disease is not long enough to be adapted to the program The possibility of a diagnosis of bipolar disorder or borderline personality disorder is systematically investigated in individual interviews with families. Teams are instructed that if these diagnoses are probable or even suspected, participants are not included in the groups. Moreover, if the patient has a possible obsessive-compulsive disorder, post-traumatic stress disorder or an addictive disorder, the family members of these patients can only be included if the symptoms favoring a diagnosis of schizophrenia are predominant.

#### Measures

Data is collected at three points, before starting Module 1, at the end of Module 1 and one year later. The two indicators measured are the suicide attempt rate (assessed before Module 1 and one year later) and the level of patient compliance as reported by families (assessed at the start and end of Module 1).

Suicide attempts: Families are asked if their ill relative attempted suicide in the 12 months prior to program participation. The occurrence of suicide after the start of the program is also studied during the evaluation one year after Module 1 by asking participants the same question, i.e. whether their loved one has made at least one suicide attempt in the last 12 months. The annual prevalence of suicide attempts (APSA) is calculated by dividing the number of participants who declared that their loved one had made at least one suicide attempt in the last 12 months by the total number of participants for whom the existence or non-existence of a suicide attempt is indicated.

Compliance: Compliance is assessed using the LSP (Live Skills Profile) (19–21). The LSP is a reference questionnaire (22) designed to assess patient functioning. It is widely used and exists in several versions with more or less items [LSP39, LSP20 (23, 24), LSP16 (25–27)]. The questionnaire is written in everyday language and is designed to enable caregivers to assess the patient’s functioning without any specific training. Compliance was associated in the different versions of the LSP with the same three items through factor analysis. The sub-score of the LSP with these three items measuring compliance gives a total score ranging from 0, corresponding to good compliance, to 9, corresponding to poor compliance.

### Taking compliance assessment biases into account when using the LSP compliance sub-score

Measuring the level of compliance is an indirect measure of treatment effectiveness (28). However, when a family caregiver or other healthcare professional estimates compliance, it is often an asymmetrical measure of treatment effectiveness. Indeed, the observation that a patient is taking his treatment correctly is more often questionable than the estimate that he is taking it incorrectly. What is more interesting than compliance is knowing whether the patient is being treated appropriately. The consequence for a patient of being insufficiently compliant is that treatment will most probably be ineffective. On the other hand, a properly compliant patient may be treated inappropriately (e.g. underdosed, or with molecules that are not clinically relevant to the patient), so we cannot be sure that the treatment is effective, despite the patient’s supposedly good compliance.

To address this problem of reliability in assessing compliance, the overall analysis of the links between suicide attempts and overall compliance scores was combined with a more targeted analysis. The latter focused solely on cases of observed poor compliance, in order to assess the effect of the program independently of drug treatment.

#### Taking into account missing data according to worst-case models

To assess the influence of missing data on the results, calculations were carried out on matched data plus data in which the final missing data were simulated using a worst-case model, based on an approach already developed in (29–31). This approach assumes that the missing data could be linked to drop-outs because the patient’s condition has worsened. This situation may result in caregiver discouragement and reduced motivation to participate. In this case, we assume that the subset of participants with missing data has no overall improvement in the indicators measured, but rather a deterioration of these indicators. On the other hand, assuming that all participants have worsened indicators would be an excessive assumption. It is more realistic to estimate a risk of worsening within a plausible and reasonable limit, rather than assuming that the worst is systematic for all missing data.

For example, for the APSA, we can assume that the rate of new suicide attempts is the same as that of the subset for which we have all the data, but that in addition, unlike this subset, all those who attempted suicide continued to do so. The equations for calculating the APSA one year before and one year after Module 1 are given in the 2nd column of **Table 1**, and the APSA incorporating the estimated missing data in the worst-case scenario in the 3rd column.

**Table 1:** APSA calculation equations.

	APSA without taking missing data into account	APSA with integration of missing data using a worst-case model
One year before M1	$\frac{\left(Nsa_{1}\;+\;Ns{a^{\prime }}_{1}\right)}{N_{1}}$	$\frac{\left(Nsa_{1}\;+\;Ns{a^{\prime }}_{1}\;+\;Nsa_{2}\;\right)}{N_{1}+N_{2}}$
One year after M1	$\frac{\left(Nsa_{1}\;+\;Nsa^{\prime }{'}_{1}\right)}{N_{1}}$	$\frac{\left(Nsa_{1}\;+\;Ns{a^{\prime \prime }}_{1}\;+\;Nsa_{2}\;+\;N_{2}*\frac{\left(Nsa'{'}_{1}\;\right)}{N_{1}}\;\right)}{N_{1}+N_{2}}$

$N_{1}$ is the number of patients without missing data at one year after M1 and $N_{2}$ the number of patients with missing data at 1 year after M1. Among the $N_{1}$ , $Nsa_{1}$ is the number of patients who attempted suicide in both the 12 months before M1 and the 12 months after M1. $Ns{a^{\prime }}_{2}$ is the number of patients who attempted suicide only 12 months before M1 (non-recurred cases) and $Nsa'{'}_{1}$ the number of those who attempted suicide only 12 months after M1 (new cases). Among the $N_{2}$ , let $Nsa_{2}$ denote the count of patients who made suicide attempts in the preceding 12 months.

In this table, $N_{1}$ be the number of patients without missing data at one year after M1 and *N*_2_ the number of patients with missing data at 1 year after M1.

Within $N_{1}$ , $Nsa_{1}$ is the number of patients who attempted suicide in both the 12 months before M1 and the 12 months after M1. $Nsa{'}_{1}\;$ is the number of patients who attempted suicide only 12 months before M1 (non-recurred cases) and $Nsa'{'}_{1}\;$ those who attempted suicide only 12 months after M1 (new cases). Among the $N_{2}$ , let $Nsa_{2}$ denote the count of patients who made suicide attempts in the preceding 12 months.

To set up worst-case compliance changes, we assume that *k*% of those with no final data actually had a worsening of their compliance, and that the others had no improvement. If the assumed value of k is too high, it risks overstating the likelihood of adverse changes in the measured indicators in cases of missing data. Yet, if a significant change is still detected after the intervention, even with this potential overestimation, it indicates that the results are robust.

Let $N_{1}$ be the number of patients without missing data at one year after M1 and $N_{2}$ the number of patients with missing data one year after M1 (See **Table 2**). Among the $N_{1}$ let $Np_{1}$ be the number of patients who have an improvement in their compliance score, $Nn_{1}$ those who have a deterioration in their compliance score. Let k be the percentage of patients whose compliance score is estimated to worsen in the subset of participants with missing data at the end.

**Table 2:** Calculation of positive and negative changes in compliance scores.

	Change in compliance scores without taking post-M1 missing data into account	Change in compliance scores with integration of post-M1 missing data using a worst-case model
positive changes	$Np_{1}$	$Np_{1}$
negative changes	$Nn_{1}$	$Nn_{1}+\;k*N_{2}$
Total changes	$Np_{1}+\;Nn_{1}$	$Np_{1}+\;Nn_{1}+\;k*N_{2}$

$Np_{1}$ is the number of patients who have an improvement in their compliance score, $and\;Nn_{1}$ those who have a deterioration in their compliance score among patients without missing data at one year after M1. $N_{2}$ the number of patients with missing data at 1 year after M1. Let k be the percentage of patients whose compliance score is estimated to worsen in the subset of participants with missing data at the end.

How to define k ? One approach is to estimate the percentage of spontaneous fluctuation in the compliance score (positive or negative) and arbitrarily presume that in the worst case all these changes are negative. We used data from another ongoing study with a test-retest of 651 caregivers at approximately three-month intervals in families about to start Profamille. The rate of patients with a change (positive or negative) before intervention was 56%.

#### Statistical analysis

Questionnaire data do not follow a normal distribution and are analyzed using non-parametric tests. The Wilcoxon or sign test is used to compare two groups with quantitative values, and the Spearman test is used to study correlations. Percentage comparisons are made using the McNemar or Chi2 test. Analysis was performed using R statistical software. The significance threshold was P<=5%.

## Results

### Characteristics of caregivers and patients

Of the 1946 caregivers who started the program, 1193 had completed questionnaires one year after the end of Module 1. This represents 39% of missing data, either due to drop-outs or to organizational problems at the centers in collecting and entering the questionnaires.

**Table 3** shows the characteristics of the caregivers and the patients in the subset with complete data at one year and in the subset with missing data at one year. Caregivers in the subset with missing data are four years younger, have relatives with an onset of illness one year earlier, with a duration of illness two years shorter, and more often live with their affected relative.

**Table 3:** Characteristics of caregivers and patients.

	Participants without missing data at 1 year post M1	Participants with missing data at 1 year post M1	p_value
Age of caregivers mean (SD) - median	59 (8.9) - 60	55 (11.2) - 56	<0.0000001.
Women caregivers	71%	70%	0.43
Patients unemployed in the last 12 months	72%	76%	0.065
patients hospitalized in the last 12 months	56%	54%	0.47
Age of patients mean (SD) - median	31 (9) - 30	29 (9) - 28	0.000007
women patients	23%	24%	0.8
Age disease onset mean (SD) - median	20 (7) - 19	19 (8) - 18	0.000006
Disease duration	25% less than or equal to 4 years 50% less than or equal to 8 years	25% less than or equal to 2,5 years 50% less than or equal to 6 years	0.05
Patients living with the caregiver	47%	56%	0.0001

#### Changes in compliance

**Figure 1:**
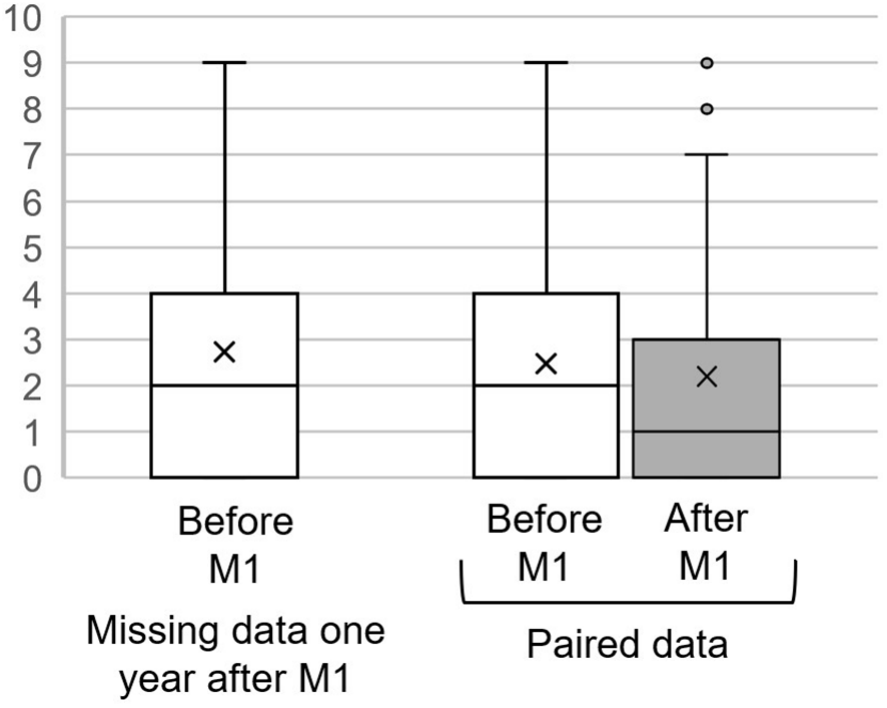
Distribution of poor compliance scores before and, when available, after the program. The y axis corresponds to the LSP compliance sub-score (the lower the score, the better the compliance). Symbol “x” is the mean, bottom of the box is the 1st quartile, center line 2nd quartile (median), top line 3rd quartile. Whiskers are the maxima and points the outliers.

The distribution of compliance scores is asymmetrical, as depicted in **Figure 1**. The bloxplot diagram shows that, based on family evaluations, a minimum of one-quarter of the patients demonstrates optimal compliance (score 0) both pre- and post-Module 1. Furthermore, at least half of the patients show a maximum initial score of 2, reducing to a maximum of 1 post-intervention. The program enhances compliance, as indicated by the two-tailed Wilcoxon test on paired data (p-value<0.000001). The average difference in scores reflects an improvement of 0.28, with a standard deviation of 2.22.

Caregivers with incomplete data records at the one-year evaluation initially reported compliance levels for their relatives that were similar to those caregivers with complete data at one year. By conducting an analysis that simulates missing data using a worst-case scenario approach, the sign test confirms that the observed improvement in compliance is statistically significant (see the final column of **Table 4**).

**Table 4:** Change of compliance scores.

	Among patients assessed at 1 year post M1	Among patients assessed at the end of M1 but not at 1 year	Simulation among patients assessed only at the beginning of M1		Integration of simulated data and non-missing data
	A	B	C		D=A+B+C
number of worsenings	301	150	56%	111	562
number of improvements	447	187	0%	0	634
Sign test bilateral p-value	0.0000001				0.040

#### Course of suicidal behavior

The average age of patients with SAs was 26.4 years, with the median age being slightly lower at 25.5 years. Of these patients, 36% were female. Both the median and mean age at the onset of their condition was 18 years. Half of the patients had a disease duration of less than seven years, and nearly half, 48%, lived with their caregiver.

**Figure 2:**
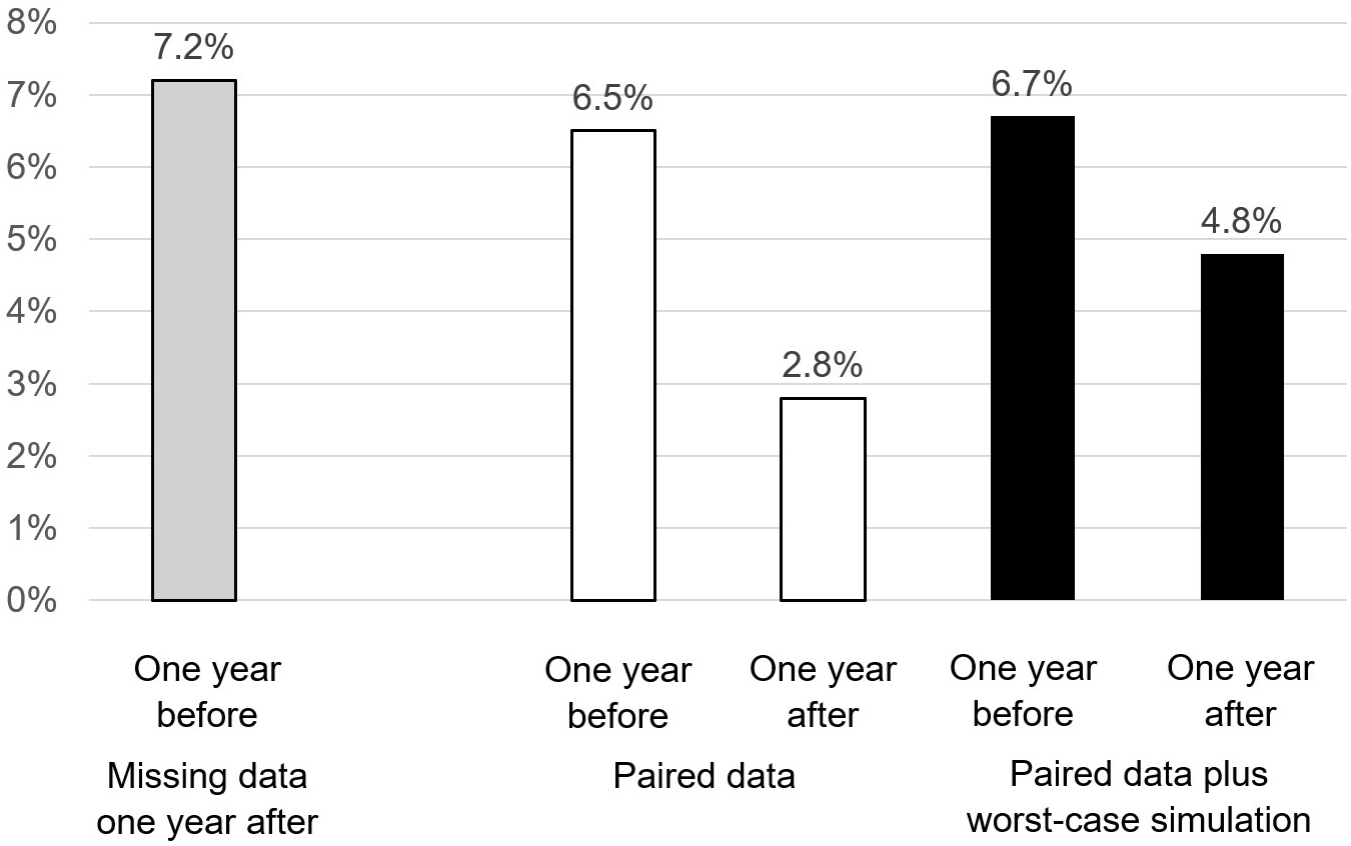
APSA one year before and one year after Profamille. The y axis corresponds to the annual prevalence of suicide attempts.

APSA was 6.5% the year before Module 1 participation and dropped to 2.8% in the subsequent year This decline is illustrated in **Figure 2**, where the central white bars depict the change, and is statistically significant as demonstrated by the Chi-square test (p-value = 0.00002). Additionally, among those who had attempted suicide in the 12 months preceding the program, 18% made another attempt in the 12 months following the completion of Module 1.

The time gap between the last SA and the beginning of the program is not precisely known but is approximated to be an average of 6 months, based on the assumption that this event is equiprobable over the preceding 12-month period. Evaluations of subsequent suicide events are conducted one year following the program’s end. This means that, typically, there’s a 6-month period prior to the program, a 6-month span for Module 1 (M1), and an additional year after M1, totaling an average follow-up duration of about 2 years from the last SA before the program. During this 2-year post-SA observation period, the suicide mortality rate was found to be 2.2%, with a 95% confidence interval of 0.3% to 7.8%.

Among those who had not committed SA in the year prior to the program, 1.4% attempted in the following year (43% male, age range [27; 44]) and 0.59% died by suicide within 2 years of their SA in the year prior to the program (50% male, age range [19; 35]).

Caregivers with missing data at one year (**Figure 2**, white bar left) initially report a slightly higher but not significantly different APSA. Worst-case simulation of missing data assumes that those who attempt suicide in the 12 months prior to their family’s participation in the program will attempt again one year later, and that 1.37% of those who didn’t attempt before will attempt after, according to the model presented above. The result (**Figure 2**, black bars on the right) shows a decrease in APSA that remains significant (Chi2 P-value=0.010). The risk ratio before/after intervention drops from 2.29 (95% CI: 1.56 to 3.39) without the missing data to 1.4 (95% CI: 1.09 to 1.83), with the addition of the missing data simulated according to the worst-case model.

#### Study of the association between compliance and suicidal behavior

There was no correlation between compliance level and APSA, nor between improved compliance and improved APSA (**Table 5**).

**Table 5:** Correlations between compliance and APSA and between change in compliance and change in APSA.

	Spearman correlation	Pairwise two-sided p-values
Initial score of lack of compliance and APSA one year before	-0.02	0.43
Final score of lack of compliance and APSA one year after	0.012	0.69
Change of compliance score and change of suicidal behavior	-0,05	0.07

At the end of Module 1, compliance had deteriorated in 268 patients (**Table 6**). In this subset, the APSA decreased from 9.0% to 2.2% (McNemar test p-value = 0.00007), aligning with the final APSA level observed across the entire patient cohort. By way of comparison, those whose compliance is improving have a final APSA of 3.4% (the difference in APSA is not significant between those whose compliance is improving and those whose compliance is worsening).

**Table 6:** Suicidal behavior in the last 12 months amongst patients with worsening treatment compliance between the beginning of M1 and the end of M1 (SA, suicide attempt, noSa, no suicide attempt).

	Before M1	
One year after M1	noSA	SA
no SA	242	20
SA	2	4

To analyze the subset with the poorest compliance and ensure adequate statistical power, the subset must be sufficiently large. In our study, this was achieved by setting the threshold for poor compliance at a score of 2 or higher, which encompassed at least one-third of the total sample. Among these patients, representing the bottom third in terms of compliance (as detailed in **Table 7**), the APSA decreased significantly by a factor greater than 2 (Chi-square test, p-value = 0.023)

**Table 7:** Suicidal behavior in the last 12 months amongst patients whose LSP lack of compliance subscore is greater than 2 (The worst third of the lack of compliance score).

Compliance in the worst third	Bad compliance and APSA Before M1	Bad compliance at the end of M1 and APSA one year after M1
No suicide attempt	371	345
Suicide attempt	28	11
Annual prevalence	7.1%	3.1%

Whitin this heterogeneous subset representing the least compliant third, as depicted in the distribution (**Figure 1**), we can identify the highly-non-compliant subgroup. This subgroup, constituting the third with the highest non-compliance scores, is defined by scores above 6. This equates to roughly 10% of the total sample. A statistical test on such a small sub-group of highly non-compliant is not relevant, but the APSA on this small sub-group after Module 1 is 2%, i.e. quite close to what is observed on the whole of the least compliant third.

**Figure 3:**
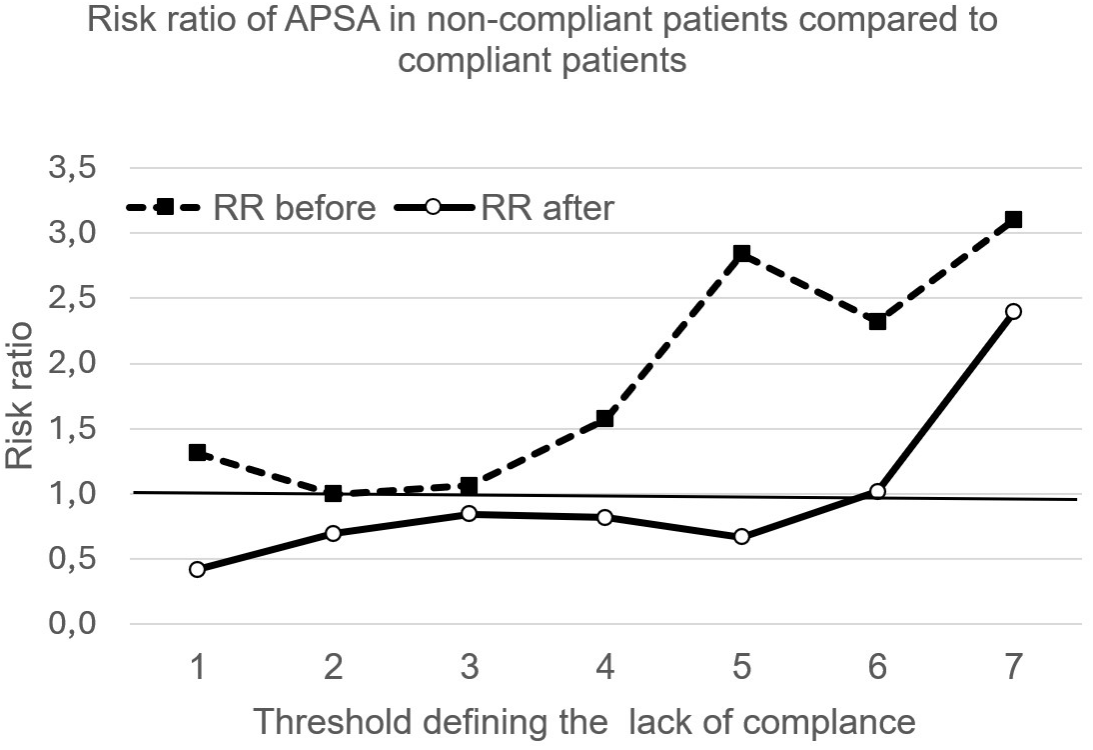
Each point is a RR of APSA of compliant patients versus APSA of non-compliant. Points with Y-axis value above 1 indicate higher risk among compliant patients, and below 1 indicate a lower risk among compliant patients.

The risk ratio (RR) of suicide attempts in compliant versus non-compliant patients may depend on the cut-off point used to categorize compliance and non-compliance. Different cut-off points have been used for this purpose. A cut-off point of “x” indicates that only patients with a score below “x” are considered compliant, and that patients with a score above “x” are considered non-compliant. For example, in **Figure 3**, for the cut-off value of 1, i.e. the value of abscissa x=1, RR before starting Profamille (dotted curve) is 1.31. This value is the ratio of the pre-Profamille APSA of non-compliant patients defined by a score below x=1 (i.e. in this example 7.7%) to the pre-Profamille APSA of compliant patients defined by a score above x=1 (i.e. in this example 5.9%). The RR for x=1 is therefore 7.7/5.9 = 1.31 (first point of the upper curve in **Figure 3**).

In the analysis of RR for APSA across varying compliance thresholds, the population sizes within compliant and non-compliant subsets were insufficient to establish statistical significance for any observed deviations from an equiprobable RR of 1. Prior to Module 1, all RRs are greater than 1 (top curve, **Figure 3**), i.e. compliant patients have a higher annual prevalence of suicide than non-compliant patients. After Module 1, we find the opposite, i.e. RR smaller than 1, except if the criterion defining compliance reaches a high level of lack of compliance, i.e. if we accept as compliant participants who reach scores of 6 or 7 (bottom curve, **Figure 3**).

## Discussion

Our aim was to show that after the participation of family caregivers in the Profamille multifamily psychoeducational program, there was an improvement in patients’ compliance compared with before their participation, to confirm that there was a reduction in patients’ APSA and to explore the possible link between improved compliance and reduced suicidal risk.

### Compliance improvement

The first point to emerge from this study is that, at the end of the program, patients’ compliance with treatment as measured by the LSP improved. This result is consistent with what has been reported about the effect of certain psychoeducational programs (11, 12). Improved compliance is usually considered to have beneficial effects for the patient (32). It is also considered to have a positive economic impact by reducing healthcare costs. In 2005, lack of compliance in schizophrenia was estimated to have cost $1479 million (33).

The absence of compliance improvement observed in other studies (18) might be attributed to differences in the methods used to assess compliance or to variations in the programs’ content and duration.

Regarding compliance measurement tools, numerous studies report that only 50% of patients with schizophrenia adhere to their treatment plan (34–37). Considering that 49% of participants in our study scored below 2 on the compliance scale before the introduction of Module M1, we can confidently suggest that a score of 2 is an indicator of satisfactory compliance, a benchmark supported by numerous studies. Post Module M1, there was an increase to 55% of patients scoring below 2, which represents a 6% improvement—a statistically significant change as indicated by a Chi-square test with a p-value of less than 0.002. Since a score below 2 on the PSL subscale detects the same proportion of compliant patients found with several other tools, the observation of improved compliance with this criterion could also be found with these other tools.

In terms of program content and duration, these factors could influence caregivers’ success in enhancing patient compliance, potentially by diminishing negative perceptions of treatment and by fostering skills that promote adherence. Such skills, including motivational techniques, often require extensive time to develop. For instance, the Profamille program’s Module M1 involves 56 hours of sessions. Shorter programs may be less effective in achieving this objective than longer ones.

### Reduction in the number of patients who attempt suicide

The second point to emerge from this study is that patients’ APSA was halved in the year following their family’s involvement in the program, compared to the year prior. This is particularly notable given that the 2011 Cochrane meta-analysis (38) did not demonstrate any impact of psychoeducational programs on suicidal risk. However, more recent evidence, including a study that monitored families over three months (9), did indicate a reduction in risk. Our results, which were initially presented and discussed in an earlier publication (10), are now backed up by a sample that has been enlarged.

The magnitude of the reduction in suicide attempts mirrors that found in a meta-analysis examining the effectiveness of cognitive-behavioral therapy directly targeting patients with a history of suicidality, compared with follow-up as usual (39). Although the study conditions are different, the fact that an “equivalent” risk reduction was obtained when the intervention targeted the family and when it targeted the patient is particularly interesting. This is especially relevant given the challenges in involving patients with lack of insight, cognitive impairments, and negative symptoms in CBT. Therefore, family-based interventions represent a valuable alternative approach that should not be neglected.

Recurrence of SA is a factor in increasing the risk of death by suicide (40), and an intervention reducing SA is also likely to reduce mortality. The rate of recurrence of SA between one year before and one year after the intervention is 18%, but this cannot be directly compared with the one-year recurrence rates cited in the literature, which range from 8% to 21% (41–43). This is because the point of measurement is not the date of the last SA, as in the literature, but the date on which the caregivers are met.

The data are also compatible with a reduction in suicide mortality, since among those who attempted suicide before the program, the death rate was 2.2% over an average follow-up of 24 months. A meta-analysis reports an average rate of 2.8% (2.2-3.5) at one year and 5.6% (3.9-7.9) over five years (44). However, the small number of people concerned in our sample (90 subjects) makes it impossible to prove this and the 95% confidence interval is too large.

### What are the links between compliance and SA?

The decrease in APSA and improvement in compliance observed after the Profamille program may suggest that it is the improvement in compliance that contributes to the decrease in APSA, which is consistent with two studies (45, 46) showing that non-compliance is associated with greater suicidal risk.

However, even though the median compliance score dropped from two to one (**Figure 1**), when the median of individual differences in compliance was analyzed, it was zero point (i.e., at least 50% had no change or even a deterioration in compliance) and the average improvement was 0.28, which appears low to have a significant effect. Moreover, the hypothesis of Profamille’s improvement on APSA from compliance appears to be contradicted by the lack of correlation found between compliance scores and suicidal behavior (**Table 5**).

The lack of correlation found between APSA and lack of compliance does not rule out the hypothesis of an effect of compliance on suicidal risk. In fact, many factors may lead to a lack of correlation, even though a causal effect exists (47, 48). The estimation of compliance by family circle members can sometimes be very different from actual compliance (49, 50). Moreover, good compliance with a treatment that is inappropriate in terms of dosage or choice of molecules can lead to the same result as poor compliance.

Suboptimal treatment prescriptions might concern between 16% (51) to 50% (52) of patients. Furthermore, the relationship between compliance and the effectiveness of treatment is not always linear. Even partially adhering to a treatment plan may not yield proportionate benefits. In some instances, anything less than full compliance could significantly diminish the treatment’s effectiveness.

Given the uncertainty surrounding the quality of what is considered good compliance (which might be overestimated or linked to inappropriate treatments), it’s more pertinent to examine the impact of the Profamille program in situations where compliance is perceived to have worsened or where poor compliance is observed. This approach is likely more reliable than relying on assessments of good compliance. However, even with this more reliable approach to compliance, we still observe two facts that contradict the hypothesis that the improvement in APSA after Profamille is mainly due to an improvement in compliance.

The first fact is that one year after the end of the program, APSA is reduced by a factor of 4 in patients whose compliance scores have deteriorated (Cf **Table 6**). A deterioration in compliance is therefore not associated with an increase in APSA. Rather, it is paradoxically associated with a notable decrease in APSA, challenging typical assumptions about the relationship between treatment compliance and suicide attempts.

The second fact is that patients who ended up in the top third for poor compliance scores (as indicated by an LSP sub-score of 3 or more) exhibited a 50% reduction in APSA compared to their baseline levels (see **Table 7**). This indicates that even among patients with low compliance, there was a significant decrease in the annual prevalence of suicide attempts.

Two objections may nevertheless be raised to the interpretation of these facts.

The first objection concerns those with worsening scores. It’s possible that for some, the decline in compliance was minimal, thus not significantly impacting APSA. A sub-group with only those with the highest aggravations would give a clearer answer, but its size in our sample is too small to have sufficient statistical power. However, in the case of those with a worsening of compliance of at least 3 points, we observe a final APSA of 2.6%, which again goes against this objection.

The second objection addresses the variability within the least compliant third of patients, whose scores range from 3 to 9. Perhaps those with really poor compliance (score above 6) have a higher APSA. This variability complicates the interpretation of the data and the relationship between compliance levels and APSA. This sub-group of highly non-compliant represents just over a third of the total number of non-compliant thirds, which does not provide sufficient statistical power. It should be noted, however, that the APSA in this small sub-group of highly non-compliant is only 2%, which counters this objection.

### Does this mean that compliance has no effect?

Prior to the program’s start, the APSA risk ratio for more compliant patients compared to less compliant ones didn’t show any notable difference, regardless of the threshold used to differentiate between them (as shown in **Figure 3**). These results are in line with some data in the literature suggesting that the protective effect of treatment on suicidal risk is not clearly established, except for clozapine (52). We have no indication of the treatments taken by patients, and as clozapine appears to be used in less than 10% of patients in France (53–55), it is likely that most would not have this treatment.

In contrast, we observe a lower relative risk of APSA after the psychoeducational program in the most compliant compared with the least compliant, except if the threshold score defining the lack of compliance is very high, i.e. if we compare the least compliant with all the others. Therefore, even if we have observed that a deterioration in compliance or a poor final compliance does not prevent the Profamille program from reducing APSA, a good level of compliance could nevertheless potentiate the effect of the program.

The limitation of our observation is that the size of the compliant and non-compliant subsets is not sufficient to have a statistical significance. Interestingly, before participating in the Profamille program, the APSA was consistently higher in the compliant subset compared to the non-compliant subset, regardless of the criteria used for defining compliance. This observation, if validated in a larger sample, could imply that adherence to treatment might paradoxically increase risks, a notion that seems counterintuitive. This is in line with some research indicating that certain antipsychotics, antidepressants, or mood stabilizers may be sometimes linked to an elevated risk of suicide (56, 57).

Another interesting result is the reversal in the relationship between compliance and APSA after the Module1: after the intervention, better compliance is systematically associated with less APSA.

The consistency of these observations before and after the program, whatever the cut-off value defining good and poor compliance, suggests that this phenomenon may not be due to chance, even if the sample size is insufficient to establish this more reliably with a statistical test. If these observations are confirmed, how are they to be interpreted?

One hypothesis might be that patients who suffer the most may, because of this suffering, both tend to look for more relief by taking the treatment better, and tend to attempt suicide. A second hypothesis could be that patients with better insight tend to take their treatment better, but better insight could also favor suicidality. However, there is no consensus on the role of insight in suicidality.

Another attractive hypothesis could be inspired by the model developed by Godlewska and Harmer (58) concerning the delay in action of antidepressants. Indeed, the treatment would create a positive change at the neuronal level in the processing of information. Subsequently, this new disposition of the nervous system would promote the learning of more positive modes of functioning or reaction through interactions with the social environment. This results in the development of new positive associations in the processes associated with learning. This model assumes that for the treatment to produce a visible behavioral effect, the social environment must be such as to enable positive interactions.

But many patients have a degraded social environment (59), with little support or enrichment, which would explain the limited effect of treatment. On the other hand, a psycho-educational program such as Profamille, which influences the family environment, helps to create a more favorable environment that not only reduces the risk of suicide independently of treatment, but also amplifies or promotes the protective effect of the treatment.

**Figure 4:**
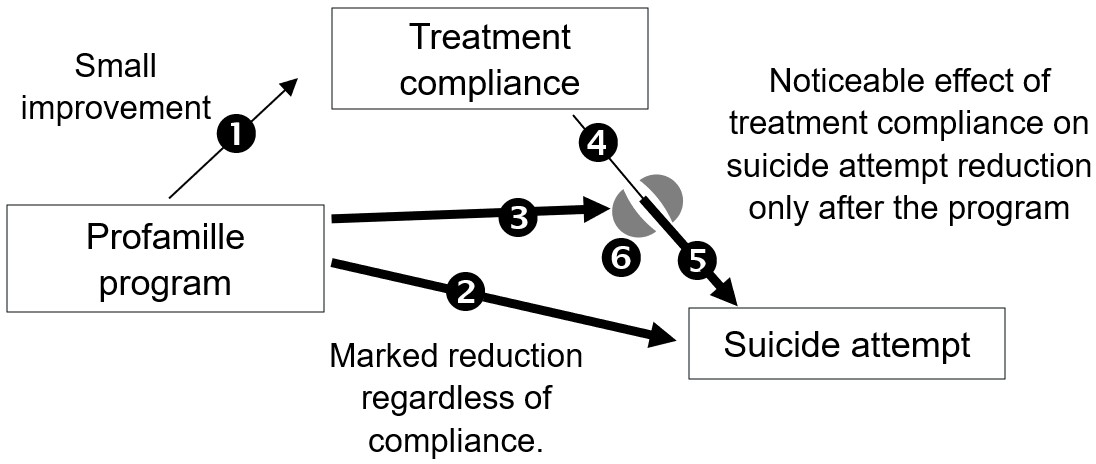
Profamille has three effects: (1) improvement of the compliance, (2) direct reduction of APSA regardless of compliance, and (3) treatment potentiation. Before the program the effect of treatment is small (4), perhaps sometimes negative, (3) on (6) Profamille creates a more supportive environment. (6) on (4) gives (5): the treatment produces its full effect under a supportive environment.

The effect of the Profamille program can be summarized in **Figure 4**.

Prior to the family’s participation in the program, if treatment has little effect, it can have side effects. The increased risk of suicide sometimes associated with treatments aimed at reducing it, such as mood regulators (60–62), antidepressants (63, 64), or antipsychotics (65, 66), can be interpreted as follows: when they fail to relieve the patient’s symptoms, the presence of significant side-effects increases distress and hence the risk of suicide.

Thus, discordant, or insufficiently clear-cut results on the effectiveness of treatments in preventing suicide in schizophrenia could stem from the variety of environments supporting the patient, with certain impoverished environments leading to a lack of effectiveness of treatments, and even in some cases to detrimental effects.

Since there are so many different environments, finding a relatively standardized, generalizable, and inexpensive intervention is no easy task. The fact that the Profamille multi-family psycho-educational program has shown clear results in improving APSA is an interesting prospect, as it is a low-cost program that is easy to implement. Over 6,000 French-speaking caregivers took part in different sub-versions of the V3 version of this program between 2010 and 2023. It has been used in a wide range of socio-economic and cultural contexts, including most departments of continental France, the French Antilles and Réunion, Belgium, Switzerland, Morocco, and Algeria. Moreover, the low cost compared with the effectiveness of this type of intervention, even outside the field of suicide reduction, and the relevance of making it a priority, was underlined in a recent meta-analysis (67), which concluded that “in contexts where there are financial constraints, family psychoeducation alone should be implemented”.

Can the results obtained on suicidal risk be generalized to any multifamily psycho-educational program? To date, there are no strong arguments for this, as programs differ in their results depending on their duration and content, as we have already shown regarding improved compliance. These results have been demonstrated with this program, but the generalization of these results to other programs with different content and structure would require specific studies. It would be interesting if the APSA were routinely assessed before and after intervention for all psycho-educational programs, in order to offer families programs with a proven reduction in suicidal risk.

### Study limitations

The limitations of this study are of two types. First, the measurements rely on declarative data, and secondly, this retrospective study lacks a control group and has a high fraction of missing data.

### Limitations in assessing the diagnosis of schizophrenia or schizophrenia spectrum disorders

All the participants come because their loved one has a severe mental disorder. Although they participate in a group which they know only involves caregivers of a patient with schizophrenia or a related disorder, and the criteria are systematically detailed, explained and verified in a one-to-one interview, we can’t rule out that some participants have a relative who suffers neither from schizophrenia nor a related disorder. Families are not asked to come with a letter confirming the diagnosis according to rigorous, standardized criteria set out by a psychiatrist following their loved one. Imposing this constraint would considerably reduce the number of family members included in the program for several reasons: 1) most psychiatric departments or private psychiatrists in France, Belgium or Switzerland do not base their diagnoses on standardized interviews. 2) many families describe difficult contacts with psychiatric departments and refusals to share information. 3) 60% of patients with schizophrenia lack insight and deny the diagnosis, which makes it difficult to obtain the patient’s consent to inform families. The way in which the diagnosis is made or suspected is not recorded by the teams, so it is not possible to compare results according to the source or probability of the diagnosis.

In the worst-case scenario, if a significant number of diagnoses were not that of schizophrenia or a related disorder, the effects of the Profamille program on suicidal risk and compliance would not be without interest.

However, it is unlikely that inappropriate inclusions will occur frequently, given the rules of exclusion, the expertise of the teams and their strong motivation to avoid including participants whose relatives have a different pathology, as this would lead to difficulties in managing a group with participants having an experience of the patient that is too different.

### Limitations related to the way suicide attempts are assessed

The reporting of SA by families may be asymmetrically biased: caregivers are unlikely to report SA they have not noticed but may also under-report SA as a result of shame, denial or because the patient is hiding his or her suicidal gesture. Participants’ guilt in reporting their relative’s attempts is reduced in the Profamille groups. Firstly, they come voluntarily to a psychoeducation group where they meet other peers and are less inclined under these conditions to hide their difficulties and feel less guilt. Secondly, it is explained to them before enrolment that they are not responsible for their loved one’s illness, and that suicide is a common behavior resulting from brain dysfunction linked to this illness. These factors reduce the risk of denial bias. Lack-of-observation bias, particularly in the case of silent SA, is possible, but it probably affects both assessment times in the same proportions, which may mitigate the impact of this bias. Moreover, it is not easy to estimate silent SA (i.e. not hospitalized and not disclosed by the patient), and this limitation is difficult to overcome.

### Limitations related to the way compliance is assessed

We mentioned earlier that the compliance scores reported in this study give an estimate of compliance similar to that found in many other studies, using other specific tools. From this point of view, the LSP sub-score provides a measure that is consistent with what is used elsewhere (34–37, 68, 69). As with the APSA measure, the compliance measure may be asymmetrically biased, with an overestimation of the latter. To reduce the consequences of this risk, part of the analysis focused on poor compliance.

### Limitations related to the absence of a control group

The fact that there is no control group raises the question of what would have been the spontaneous evolution without intervention. Regarding APSA, it is shown in (10) with a subset of the current cohort that its decline after intervention is much greater than the spontaneous decline that can be observed over time.

Concerning the compliances score, based on the above-mentioned data from 651 participants who completed the LSP questionnaire on 2 successive occasions at an interval of 3 months prior to the intervention (test-retest), the difference was not significant (p= 0.056). However, perhaps a larger population would have led to a different conclusion. The fact that no improvement in compliance was observed with other psychoeducational programs (18) may also suggest that compliance does not improve spontaneously. However, the observation cited was based on a small population.

The retrospective nature of the study may mask recruitment bias towards people who are more sensitive to the efficacy of the intervention, or who may spontaneously show a more favorable evolution of the indicators measured. It is not possible to rule out this hypothesis, but the majority of participants initially showed signs of exhaustion and depression, which they reported as persistent. There are no strong arguments to suggest that their chronic depressive state, linked to persistent environmental stress, could have improved spontaneously. However, at the end of the program, several studies have shown a clear improvement in the participants’ mood (29, 70, 71). Although mood improvement is a different indicator from those studied here, this suggests that the program has positive effects unlikely to be linked to spontaneous evolution. This supports the idea that the improvement in the indicators studied in this cohort could not be attributed to spontaneous improvement.

### Limitations due to missing data

Finally, another limitation of the study is the percentage of missing data. These are mainly organizational problems leading to a lack of rigor in collecting and entering data, with the various teams interviewed reporting participants who continued to take part in the group and were doing well, but whose questionnaires had not been retrieved or had sometimes been lost.

Although caregivers and patients are slightly younger in the subset of participants with missing data, and more often live under the same roof, this age difference is small, and the influence of these factors is not obvious to judge. The values of the studied APSA and initial compliances parameters are not significantly different from those for which we have data at one year (**Figures 1**, **2**). This lack of obvious difference between those with missing data and those without is also noted in other studies on other parameters and with other data, but with the same Profamille program (10, 29).

On the other hand, simulations of missing data using the worst-case model show that the improvement in compliance and the decrease in APSA remain significant. Although we cannot rule out the possibility that the worst-case simulation model underestimates the number of patients lost to follow-up due to worsening, the assumptions chosen to estimate these risks are fairly conservative and give robustness to the results found.

## Conclusion

This study shows that the Profamille psychoeducational program in its V3.2 version improved treatment compliance and reduced APSA. This is observed even when replacing missing data with simulated results unfavorable to this conclusion according to a worst-case model. This, added to the size of the study population, which exceeds one thousand subjects, suggests a certain robustness of this conclusion. However, it is not this increase in compliance that explains the drop in APSA. There is a decline in APSA even in cases of deteriorating compliance, or even in cases of low compliance, which should encourage the referral of many families to such a program.

This study also suggests that compliance may have little effect on suicidal risk in an insufficiently supportive environment, but that the protective contribution of treatment on suicidal risk is revealed after family psychoeducation. This suggests that prescribing medication alone to reduce the risk of suicide in schizophrenia is a strategy that may be lacking in effectiveness if it is not systematically combined with psychoeducation of the family using an appropriate program. Failure to refer families to such programs could therefore be seen as a loss of chance, especially as this type of intervention is inexpensive and easy to implement.

## Data availability statement

The data analyzed in this study is subject to the following licenses/restrictions: collaborative study with authors. Requests to access these datasets should be directed to yann.hode@yahoo.fr.

## Ethics statement

Ethical approval was not required for the study involving humans in accordance with the local legislation and institutional requirements. Written informed consent to participate in this study was not required from the participants or the participants’ legal guardians/next of kin in accordance with the national legislation and the institutional requirements.

## Author contributions

YH: Conceptualization, Formal analysis, Methodology, Writing – original draft, Data curation, Project administration, Software, Supervision, Validation, Writing – review & editing. RP: Formal analysis, Investigation, Writing – original draft, Writing – review & editing. WH: Investigation, Methodology, Writing – review & editing. NG-B: Investigation, Writing – review & editing. JA: Investigation, Writing – review & editing. MB: Investigation, Writing – review & editing. M-CB: Investigation, Writing – review & editing. IC: Investigation, Writing – review & editing. OC: Investigation, Writing – review & editing. AM: Investigation, Writing – review & editing. CR: Investigation, Writing – review & editing. SL: Investigation, Writing – review & editing. DW: Investigation, Writing – review & editing.
